# Overexpression of *AtPROPEP6* enhances *Arabidopsis thaliana* resistance to Southern root-knot nematode *Meloidogyne incognita*

**DOI:** 10.1080/15592324.2026.2624239

**Published:** 2026-02-08

**Authors:** Payal Sanadhya, Kallahan Minor, Jiamei Li, Suxing Liu, Alexander Bucksch, Alisa Huffaker, Joanna Kud, Fiona L. Goggin

**Affiliations:** a Department of Entomology and Plant Pathology, University of Arkansas, Fayetteville, AR, US; b School of Plant Sciences, University of Arizona, Tucson, AZ, US; c Department of Cell and Developmental Biology, University of California San Diego, La Jolla, CA, US

**Keywords:** Plant elicitor peptide, *AtPROPEP6*, *Meloidogyne incognita*, plant defenses, root-knot nematode

## Abstract

Plant elicitor peptides (Peps), derived from PROPEP protein precursors, are stress-induced signaling molecules that enhance plant immunity. While previous studies of Pep-mediated immune signaling in *Arabidopsis thaliana* have focused on the roles of *AtPROPEP1–3* genes in bacterial and fungal resistance, this study identifies the *AtPROPEP6* gene as a contributor to defense against the Southern root-knot nematode (*Meloidogyne incognita)*. *In silico* promoter analysis revealed enrichment of W box motifs, suggesting potential regulation by WRKY transcription factors associated with plant immune responses. Unlike other *PROPEP* gene family members, *AtPROPEP6* shows specific upregulation in response to ascr#18, a nematode-derived molecular pattern, but not to other pathogen elicitors. Transgenic constitutive overexpression of *AtPROPEP6* in *A. thaliana* significantly reduced gall formation and total nematode numbers and delayed nematode development. These phenotypes correlated with *AtPROPEP6* transcript abundance in three independent transgenic lines and were accompanied by elevated basal *AtPR1a* expression. Although *AtPROPEP6*-overexpressing plants exhibited shorter roots, the extent of root length reduction did not align with transgene expression levels, and the number of root tips available for infection remained unchanged. Our findings expand the repertoire of defense-associated *A. thaliana PROPEP*s beyond *AtPROPEP1–3* and identify *AtPROPEP6* as a paralog contributing to plant defense against nematodes.

## Introduction

Plant elicitor peptides (Peps) are endogenous molecules released upon pathogen attack or physical injury, commonly referred to as damage- or danger-associated molecular patterns (DAMPs).^1^^,^^2^ Peps were originally discovered in *Arabidopsis thaliana*, but are broadly conserved across both monocots and dicots, including key crops such as maize, soybean, tomato, potato, rice, sorghum, and canola.^3^^,^^4^ In their bioactive form, Peps are only 23 to 36 amino acids in length, however they originate from the C-terminal region of larger precursor proteins known as PROPEPs. Individual PROPEPs localize either in the cytoplasm or the tonoplast and are cleaved by the metacaspases upon stress stimulus.^3^^,^^5^ Then, Peps are actively or passively released to the apoplast, where they are perceived by transmembrane Pep elicitor receptors (PEPRs) belonging to the LRR-RLK family (Leucine-rich repeat receptor-like kinase), which initiate defense signaling cascades.^6^^,^^7^ Interestingly, Pep treatments elicit more robust immune responses in roots than the typical pathogen-associated molecular patterns (PAMPs), such as flagellin and chitin.^8^

Several studies have shown that Peps play a role in activating plant defenses against both the above- and belowground biotic stresses, including microbes, insects, and nematodes. In *A. thaliana*, overexpression of *AtPROPEP1* reduced the necrotrophic oomycete *Pythium irregulare* infection,^3^ and foliar application of *At*Pep2/3 imparted resistance to the hemibiotrophic bacterial pathogen, *Pseudomonas syringae pv. tomato* DC3000.^7^ Conversely, loss of function of both Pep receptor genes (*AtPEPR1* and *AtPEPR2*) increased susceptibility to foliar herbivory by the *Spodoptera littoralis.*^9^ In crops, *Zm*Pep1 pretreatment protected maize from the fungal pathogens *Colletotrichum graminicola* and *Cochliobolus heterostrophus,*^10^ and soybean seed treatments with *Gm*Pep1, *Gm*Pep2, and *Gm*Pep3 reduced the soybean cyst nematode (SCN: *Heterodera glycines*) and the Southern root-knot nematode (SRKN; *Meloidogyne incognita)* infection.^11^^,^^12^

In *A. thaliana*, the genomic organization and expression patterns of the eight *PROPEP* genes (*AtPROPEP1–AtPROPEP8 genes*, encoding *At*Pep1-*At*Pep8 peptides) suggest functional differences between those paralogous. *AtPROPEP2/1/3* and *AtPROPEP8/7/4/5* form two distinct gene clusters on chromosome 5,^3^^,^^13^ and wounding or pathogen elicitors activate transcription of *AtPROPEP2/1/3* but not *AtPROPEP8/7/4/5*. Moreover, within these groupings, individual *AtPROPEP* genes display distinct spatial and temporal expression patterns.^13^ *At*Peps also differ in their ability to stimulate mutants with impairments in *AtPEPR1* or *AtPEPR2*, implying different binding affinities for the two receptors.^13^ Furthermore, *At*Peps function appears to extend beyond regulating plant immunity. A mutant with loss of function of *AtPROPEP2* displayed a constitutive reduction in root hair growth, indicating a role for *At*PEP2 in normal root development independent of stress responses.^14^

To date, relatively little is known about the role of *AtPROPEP6*. Whereas all other *AtPROPEP* genes are clustered on chromosome 5, *AtPROPEP6* is a solitary member located on chromosome 2.^3^^,^^13^ Studies of the activity of all eight *AtPROPEP* genes in roots indicated that constitutive patterns of *AtPROPEP6* are most similar to *AtPROPEP1* and *AtPROPEP2*, with strong expression throughout root tissues and localization in the tonoplast.^13^^,^^14^ However, little information is available about *AtPROPEP6* stress responses because most previous analyzes of the responsiveness of *AtPROPEP* family to stimuli have excluded this paralog.^13^^,^^15^ Based on this evidence for multifunctionality and potential functional diversification among Peps, it is important to characterize the impacts of each family member on both growth and defense.

To address this knowledge gap, the objective of this study was to determine if *AtPROPEP6* plays a role in *A. thaliana* defense responses to *M. incognita* infection and to assess whether *AtPROPEP6* contributes to host resistance independently of changes in root traits. Unlike other *PROPEP* genes, *AtPROPEP6* is specifically induced by the nematode-derived signal ascr#18. Transgenic constitutive overexpression of *AtPROPEP6* in *A. thaliana* enhanced resistance by reducing early nematode root penetration, nematode development, and root galling, effects that correlated with elevated *AtPR1a* expression rather than changes in root length. Our data support a role for *AtPROPEP6* in modulating Pep-mediated immunity during nematode infection.

## Materials and methods

### Nematode cultures

*Meloidogyne incognita* was maintained on greenhouse grown tomato (*Solanum lycopersicum cv. Rutgers*), and nematode eggs were extracted from infected roots using 0.05% (v/v) sodium hypochlorite (NaOCl), followed by sucrose flotation.^16^ Eggs were surface sterilized by sequential treatment with 0.7% (v/v) streptomycin for 10 min and 0.01% (w/v) mercuric chloride for 10 min, followed by thorough rinsing with sterile distilled water. Sterile eggs were collected on 25-μm sieves and incubated in 0.01 M MES buffer (Sigma-Aldrich) in the dark for 3 days to allow hatching.

### In silico promoter analysis

The 1,743 kb region upstream from *AtPROPEP6* translation start site, which represents the entire intragenic region up to the next 5’ open reading frame, was used for cis-regulatory elements (CREs) identification with NewPLACE.^17^

### *AtPROPEP6* overexpression lines

The *35S:AtPROPEP6-oe* lines were generated using the same methods as we previously described for *35S:AtPROPEP1-oe* lines.^3^ Briefly, the expression cassette containing the *AtPROPEP6* genomic sequence under the control of the cauliflower mosaic virus 35S promoter was constructed using the pART7/pBART binary vector system.^18^ *A. thaliana* (ecotype Columbia-0, Col-0) plants were transformed via the *Agrobacterium tumefaciens*-mediated floral dip method,^19^ and transgenic *A. thaliana* seedlings were selected on Murashige and Skoog media (MS; PhytoTech Labs, Lenexa, KS, USA) containing BASTA (10 μg/ml). The transgenic status of each line was confirmed both by BASTA selection and by PCR with pART F (5’ CTT CGC AAG ACC CTT CCT CTA 3’) and pART R (5’ CAT AGG CGT CTC GCA TAT CTC 3’) primers to amplify the inserted T-DNA region.^20^ *AtPROPEP6* expression was compared in more than eight independent events using semi-quantitative RT-PCR as described before,^20^ and those with at least ten-fold higher expression than untransformed controls were selected for further propagation and analysis. Events were selfed, and segregation was tracked in subsequent generations using both BASTA selection and pART primers, through which we developed three non-segregating lines, each from a different independent event (*35S:AtPROPEP6:2-oe*, *35S:AtPROPEP6:6-oe*, and *35S:AtPROPEP6:8-oe*). Lines were passed through at least three generations before use for this study. An overview of the analyzes performed on these lines is presented in Figure S1.

### RNA extraction and qRT-PCR

Rosette tissues from wild-type Col-0 and transgenic *A. thaliana 35S:AtPROPEP6-oe* plants cultivated in potting mix were collected 14 days after transfer to soil and flash-frozen in liquid nitrogen. Total RNA was extracted from the leaf tissue using RNeasy Plant Mini Kit (Qiagen, Germany), and mRNA was converted to cDNA with SuperScript III reverse transcriptase and oligo dT primers (Invitrogen, Carlsbad, CA, USA). Gene expression was determined by qRT-PCR (initial denaturation 95 °C for 5 min, followed by 40 cycles of 95°C/15 s, primer specific °C/30 s, and 72°C/30 s. A melting curve analysis was performed at the end of the PCR run over the range 60–95°C, increasing the temperature by 0.5 °C every 10 s. Gene expression was calculated using the ^ΔΔ^Ct method with values normalized to the geometric mean of expression levels of three stable endogenous reference genes: *Actin2* (At3G18780), *EF1A* (At5G60390), and *F-Box* (At5g15710).^21‐25^ All primers, their corresponding annealing temperatures, and amplification efficiencies are listed in Table S1.

### *In vitro M. incognita* infection assays

*A. thaliana* seeds were surface-sterilized by 5 min treatment in 70% ethanol, followed by five sterile distilled water washes, then 10 min in a 1.4% aqueous NaOCl solution. The seeds were then washed five times once again with sterile distilled water. Sterilized *A. thaliana* seeds were germinated on the Murashige and Skoog (MS; PhytoTech Labs, Lenexa, KS, USA) medium supplemented with 2% (w/v) sucrose and 0.8% Gelrite (Sigma-Aldrich, St. Louis, MO, USA). Germinated seedlings were transferred to individual Petri Dish plates after 7 days and plates were then grown vertically in a growth chamber (22 °C; 12 hrs. light, 12 hrs. dark). At 14 days after germination, each seedling was inoculated with either 300 or 500 freshly hatched, sterile *M. incognita* J2s, applied near the root zone for nematode penetration and galling assay, respectively. Each experiment had at least 8 biological replicates (n ≥ 8). Roots were gently harvested at either 3- or 28-days post-inoculation (dpi), stained with acid fuchsin,^26^ and observed under the stereomicroscope (Nikon SMZ800 microscope, Nikon Metrology Inc.) to visualize different nematode development stages and galls (Figure S1). Total female counts reported the number of both mature and young females, with examples of these stages shown in Figure S1d. Each experiment was repeated twice and included at least eight biological replicates per transgenic line.

### Root morphology analysis

*A. thaliana* seeds were germinated and seedlings grown in tissue culture conditions on MS media as described earlier. Roots from 14-day-old plants were scanned using an EPSON Perfection V30 scanner (Epson America Inc., USA), and primary root length as well as the number of secondary roots were quantified with ImageJ software.^27^ The experiment was repeated twice with *n* = 10.

### Shoot phenotypic analysis and bolting

*A. thaliana* seeds were germinated in tissue culture conditions on MS media for 7 days and then moved to pots with Sungro LC1 Sunshine potting mix (Sungro Horticulture, Belevue, WA) supplemented with Osmocote Plus 15-9-12 slow-release fertilizer (Scotts-MiracleGro Company, Marysville, OH). Plants were maintained in a growth chamber (22 °C, 12 hrs. light, 12 hrs. dark). After 14 days, plants were photographed from above to capture the area of the rosette; leaf area was calculated from images using the SMART tool.^28^ This timepoint was selected because it was the last point at which the plants could be imaged from above without extensive interference from the developing inflorescences. Bolting, defined as the transition from vegetative growth to reproductive development marked by elongation of the inflorescence stem, was assessed by counting the number of plants that had bolted (i.e., produced visible inflorescences) 21 days after transfer to soil. The experiment was repeated twice with *n* = 10.

## Results

### In silico promoter analysis of *AtPROPEP6* and its expression in response to ascr#18

**Figure 1.**
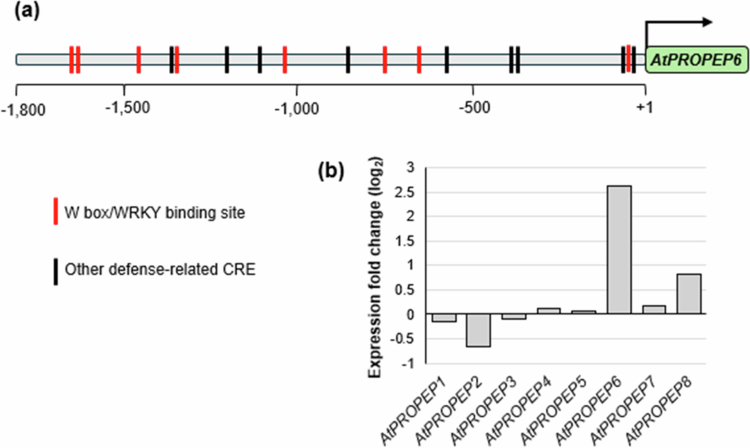
Transcriptional regulation of *AtPROPEP6* expression suggests an involvement in plant defenses**.** (a) Defense-related Cis-Regulatory Elements (CREs) in the promoter of the *AtPROPEP6* gene. The positional distribution of predicted CREs is shown as vertical color-coded bars for individual binding sites (see also Table S2). Red–W box/WRKY binding site; black - other defense-related CREs. (b) Log_2_ fold change of *AtPROPEPs* expression in response to ascr#18 treatment. The expression pattern data were obtained from the published RNAseq analysis of *Arabidopsis* genes induced by ascr#18 treatment (1 µM ascr#18 treatment for 6 hours) -NCBI Bio Project Accession PRJNA550121. Numbers represent averages of 4 biological replicates, *p*-value < 0.05, FDR < 0.05.^30^

To address the relative lack of knowledge of *AtPROPEP6* function, we first conducted an *in silico* promoter analysis for this overlooked family member. The 1,743 kb region upstream from *AtPROPEP6* translation start site, which represents the entire intragenic region up to the next 5’ open reading frame, was used for cis-regulatory elements (CREs) identification with NewPLACE.^17^ The analysis revealed 19 defense-associated cis-regulatory elements (CREs), which accounted for approximately 8% of the total predicted transcription factor binding motifs (Figure S2). Notably, within that subset of immunity-linked CREs, we uncovered 8 binding sites for putative WRKY transcription factors (W box) (Figure 1a, Table S2). Given that prior *in silico* and *in vivo* analyzes of defense-related *AtPROPEP1–3* promoters demonstrated that W boxes are critical for mediating their transcriptional upregulation in response to elicitor treatments,^29^ this finding suggests a potential role for *AtPROPEP6* in plant defense. Additionally, our analysis of previously published RNAseq data obtained from *A. thaliana* Col-0 treated with ascr#18, a nematode-associated molecular pattern (NAMP), showed strong 6-fold induction of *AtPROPEP6* compared with other *AtPROPEPs* displaying only weak or no induction at all ^30^ (Figure 1b). Building on these findings, we hypothesize that *AtPROPEP6* may play an important role in plant defenses to phytonematodes.

### Overexpression of *AtPROPEP6* uniquely impacts the expression pattern of marker genes

**Figure 2.**
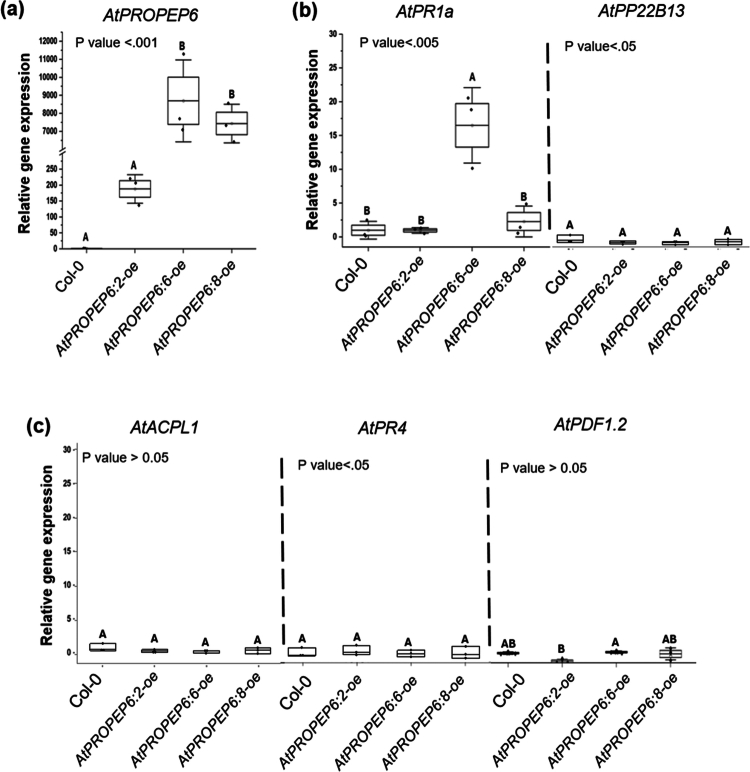
*AtPROPEP6* overexpression increased the basal expression of the *AtPR1a* marker gene. The relative expression of (a) *AtPROPEP6*, (b) SA marker genes (*AtPRa1, AtPP22B13*), and (c) JA marker genes (*AtACPL1, AtPR4, AtPDF1.2*) in homozygous *AtPROPEP6-oe* lines (6:2, 6:6, and 6:8) was determined by qRT-PCR. Results are presented as the fold change in relative basal expression compared to non-transgenic control (Col-0). Different letters indicate statistically significant differences among treatments (one-way ANOVA followed by Tukey’s post hoc test; *α* = 0.05; *n* = 3).

To assess the effects of *AtPROPEP6* overexpression on nematode infection, we generated multiple independent overexpression events and selected three homozygous, single-insertion lines exhibiting varying levels of *AtPROPEP6* transcript abundance for subsequent experiments. The *AtPROPEP6:6-oe* line had the highest transgene transcript accumulation, *AtPROPEP6:8-oe* showed moderate levels, and *AtPROPEP6:2-oe* had the lowest expression (Figure 2a). Previous studies have revealed that overexpression of *AtPROPEP1* and *AtPROPEP2* upregulates the expression of several defense-related genes, including salicylic acid (SA) marker genes (*AtPR1a* and *AtPP22B13*) and the ethylene and jasmonic acid (ET/JA) marker genes (*AtPDF1.2, AtPR4, AtACPLA1).*^31^^,^^32^ Interestingly, *AtPR1a* basal expression was significantly higher in the *AtPROPEP6:6-oe* line with the highest *AtPROPEP6* transcript level. In contrast, the expression of other tested marker genes remained unchanged (Figure 2b and 2c). These results show that *AtPep6* is capable of uniquely priming plant defenses before biotic challenge.

### Overexpression of *AtPROPEP6* reduces *M. incognita* ability to penetrate roots and delays nematode development

**Figure 3.**
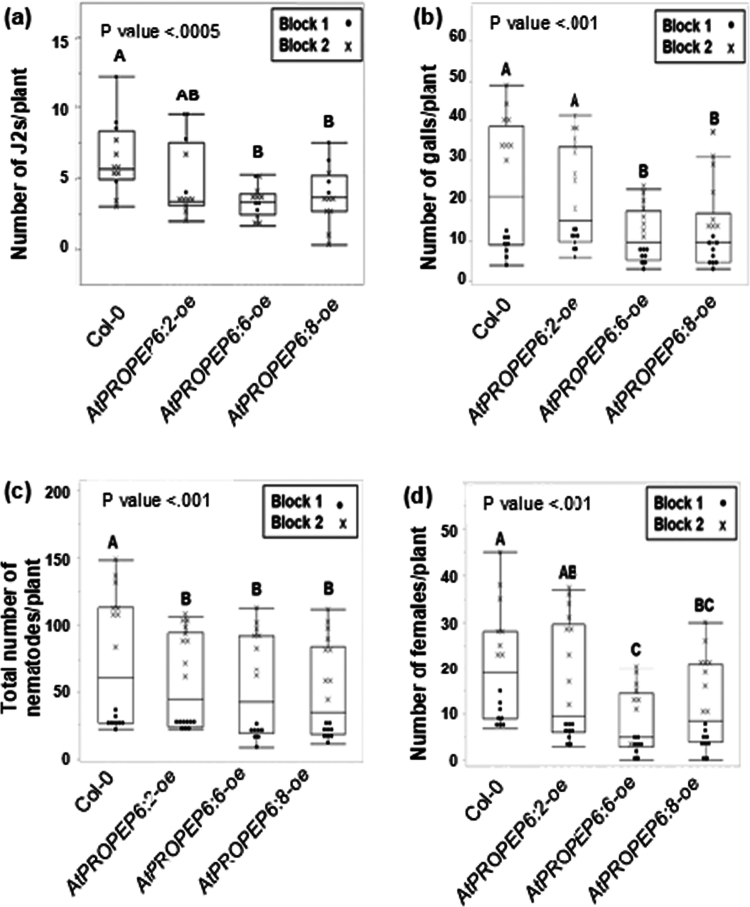
*AtPROPEP6* overexpression reduces *Arabidopsis* susceptibility to *M. incognita* infection**.** (a) Root penetration assay scored at 3 dpi, and (b*–*d) galling and nematode development assay scored at 28 dpi (including (b) number of galls, (c) total nematode count, and (d) number of females). Nematode infection assays were performed on three transgenic *AtPROPEP6-oe* lines (6:2, 6:6, and 6:8) and wild-type Col-0 as described in Figure S1. Data points were obtained from two independent experiments (Blocks 1 and 2) with a total of *n* = 16 (a) and *n* = 8 (b*–*d) for each line per experiment. Different letters indicate statistically different results between treatments (one-way ANOVA followed by Tukey’s post hoc test *p* < 0.05).

To evaluate the impact of the *AtPROPEP6* overexpression on both early and late stages of the *M. incognita* infection process, we examined nematode penetration at 3 dpi and nematode development at 28 dpi. At 3 dpi, significantly fewer juveniles had entered *AtPROPEP6:6-oe* and *AtPROPEP6:8-oe* than the untransformed control (Col-0), and nematode numbers on *AtPROPEP6:2-oe* were intermediate but not statistically different compared with the control (Figure 3a). Consistently, *AtPROPEP6:6-oe* and *AtPROPEP6:8-oe* also exhibited fewer galls at 28 dpi, a measure of root damage by nematode (Figure 3b). In addition to the total nematode count (all life stages) at 28 dpi (Figure 3c), we also quantified the number of females to assess effects on nematode development (Figure 3d; see Figure S1 for details). While total nematode numbers were reduced by 21%, 27%, and 32% in all overexpression lines compared to Col-0 control, the number of females was decreased more substantially by approximately 20%, 58%, and 45% in the *AtPROPEP6:2-oe*, *AtPROPEP6:6-oe*, and *AtPROPEP6:8-oe* lines, respectively. These results suggest that *AtPROPEP6* overexpression delays nematode maturation, with the strongest effect observed in *AtPROPEP6:6*-oe, which also exhibited the highest accumulation of transgene transcripts. Collectively, these findings demonstrate that *AtPROPEP6* overexpression not only limits nematode entry but also impairs their development within host roots.

### *AtPROPEP6* overexpression influences plant morphology and bolting

**Figure 4.**
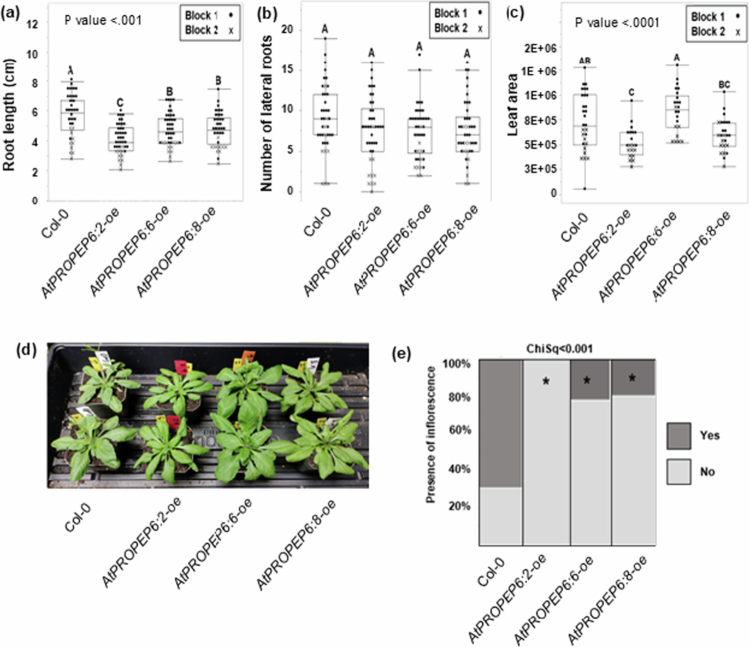
Overexpression of *AtPROPEP6* impacts root morphology and bolting. Belowground morphological measurements included (a) root length and (b) number of lateral roots in 14-day-old plants. Aboveground traits were recorded as (c*–*d) leaf area and (e) bolting at 21 days. For (a*–*c), data points were obtained from two independent experiments (Blocks 1 and 2). Different letters indicate significant differences (one-way ANOVA with Tukey's HSD, *P* < 0.05, *n* = 10). For (e), lines marked with an asterisk are significantly different from Col-0 control based on Chi-square test with a total of *n* = 10 for each line per experiment (*p* < 0.001).

In light of the considerable evidence supporting the idea that, *PROPEP* genes also influence plant growth and development, we next investigated whether overexpression of *AtPROPEP6* results in changes to plant morphology. To assess belowground growth, which could potentially impact nematode infection, we measured the length of the primary root and the number of lateral roots in 14-day-old seedlings, the growth stage used for nematode inoculation in our infection assays. Overexpression of *AtPROPEP6* resulted in significantly shorter primary roots across all three tested lines, although root length did not correlate with transgene expression. The *AtPROPEP6:2-oe* line, which had the lowest transcript levels, exhibited the most pronounced root growth inhibition, while *AtPROPEP6:6-oe* (highest expression) and *AtPROPEP6:8-oe* (intermediate expression) showed milder effects (Figure 4a and Figure S4). Despite differences in the length of the primary root, there were no significant differences in the number of lateral roots between wild-type and transgenic lines (Figure 4b), indicating that all lines had comparable numbers of root tips available for *M. incognita* infection. Lastly, because *AtPROPEP1-oe* and *AtPROPEP2-oe* were previously linked to increased aboveground growth, we also evaluated leaf area and weight at 14 days, as well as bolting at 21 days post transfer to soil. While *AtPROPEP6:2-oe* and *AtPROPEP6:8-oe* had reduced leaf area and weight compared to wild-type plants, *AtPROPEP6:6-oe* did not significantly differ from to wild-type control (Figure 4c-d, Fig S5); thus, once again, the effects of *AtPROPEP6* overexpression on growth were not directly correlated with transgene expression levels or with nematode resistance.

## Discussion

Peps are conserved proteinaceous signaling molecules that play important roles in plant stress responses and the regulation of both growth and development. In *A. thaliana*, the eight *PROPEP* paralogs differ in their genomic organization, expression patterns, and affinity to bind PEPR receptors, strongly supporting the notion that, despite some functional redundancy, substantial functional divergence exists among family members. Here, we focus on the previously largely uncharacterized *AtPROPEP6*, particularly in the context of its role in plant immune responses to *M. incognita*. Our findings expand the known set of defense-associated *A. thaliana PROPEPs* beyond *AtPROPEP1-3*, identifying *AtPROPEP6* as a paralog involved in plant defenses to nematodes.

The available literature describes the basal expression of *AtPROPEP6* as being most closely related to *AtPROPEP1* and *AtPROPEP2*, with strong activity throughout root tissues,^13^^,^^14^ but comparatively little is known about stress-inducible regulation of this paralog. Transcriptional control of several *AtPROPEP* family members has been linked to WRKY transcription factors. In particular, Logemann et al. demonstrated that W-box cis-elements in the promoters of *AtPROPEP2* and *AtPROPEP3* are directly bound by *At*WRKY33 upon defense elicitor treatments.^29^ *At*WRKY33 is a key immune-related transcription factor involved in multiple defense pathways and has been shown to positively contribute to *A. thaliana* resistance to cyst nematodes; ^33^ however, its role in immunity against root-knot nematodes has not been directly tested. Nevertheless, other WRKY transcription factors are also implicated in plant-nematode interactions. For example, *AtWRKY11* is induced by *M. incognita* infection and functions as a positive regulator of defenses against both root-knot and cyst nematodes.^34^ Given the enrichment of W-box motifs in the *AtPROPEP6* promoter, we speculate that WRKY-mediated transcriptional regulation is a plausible mechanism controlling *AtPROPEP6* expression during nematode infection and warrants further investigation.

Unlike *AtPROPEP1–3, AtPROPEP6* is uniquely responsive to ascr#18 treatment, but it is not induced by methyl jasmonate, methyl salicylate, or other pathogen elicitors, including a necrosis-inducing *Phytophthora* protein (NPP1), *Pseudomonas* harpin protein HrpZ, or the most conserved domain of bacterial flagellin protein (flg22).^3^^,^^30^ This unique expression pattern suggests a potential role in plant-nematode interactions. Our analysis of the *AtPROPEP6* overexpression lines revealed reduced susceptibility to *M. incognita*, manifested by a lower early penetration rate, which correlated with fewer galls and diminished nematode development at later infection stages. While total nematode numbers were reduced across all three lines, the decline in female counts was more pronounced, indicating that *AtPROPEP6* expression interferes not only with host entry but also with nematode maturation. The strongest suppression of female development was observed in *AtPROPEP6:6-oe*, consistent with its highest transgene expression, suggesting a potential dose-dependent effect. Together, these results identify *AtPROPEP6* as a contributor to nematode resistance that limits parasitic success within host roots at more than one stage of infection. However, the precise mechanisms underlying this effect remain to be elucidated.

Our data show that constitutive overexpression of *AtPROPEP6* selectively elevates basal accumulation of *AtPR1a* transcripts, while having no significant effect on the other tested markers previously reported to be induced by defense-related *At*Pep1-3.^31^^,^^32^ Specifically, the absence of *AtPDF1.2* induction in *AtPROPEP6* overexpression lines contrasts with the elevated transcript accumulation of this marker gene observed upon overexpression of *AtPROPEP1* or *AtPROPEP2*, further supporting the notion that *AtPROPEP6* represents a functionally distinct member of the *PROPEP* family.^31^ This expression profile aligns with earlier studies showing that *M. incognita* infection of *A. thaliana* roots strongly induces *SA-*associated *PR* genes, including *AtPR1a,* whereas ET/JA-associated *PR* genes such as *AtPR4* show no transcriptional response.^35^ *M. incognita* is a sedentary endoparasite that establishes a long-term biotrophic relationship with the host plant, making activation of SA-mediated defense pathways consistent with the nature of this interaction.^32^ Notably, *AtPR1a* appears to play an uniquely important role in nematode defense, as it was the only *PR* gene among RKN-induced SA markers (*AtPR1, AtPR2, and AtPR5)* whose constitutive overexpression in transgenic *A. thaliana* significantly suppressed *M. incognita* galling.^35^

Interestingly, exogenous application of *At*Pep1 caused dose-responsive, *At*PEPR2-dependent inhibition of root elongation.^8^^,^^36^^,^^37^ On the other hand, constitutive overexpression of *AtPROPEP1* and *AtPROPEP2* resulted in visibly larger root and shoot biomass,^3^ and the *AtPEPR2* receptor mutant, *atpepr2*, displayed a shorter root phenotype than wild-type plants.^38^ Thus, the relationship between Pep dosage and growth responses may be complex, given the conflicting reports on the effects of *PROPEP* overexpression or ectopic Pep treatment on plant growth. In this study, while *AtPROPEP6-oe* lines display slightly shorter primary roots than Col-0 control, this trait did not correlate with transgene expression in a similar manner to *AtPR1a* gene induction and impact on *M. incognita* infection. Unlike cyst nematodes, which can penetrate roots at variable positions, root-knot nematodes preferentially enter roots just behind the root tip, within the meristematic and elongation zones.^39^^,^^40^ Root tips are particularly permissive to root-knot nematode entry because these tissues exhibit minimal lignification, resulting in comparatively thinner and less rigid cell walls than those of mature taproot tissues.^41^ Consequently, differences in overall root length are unlikely to substantially influence infection rates, especially given that root tip availability remained unchanged in the *AtPROPEP6* overexpression lines. Additionally, the observed reductions in leaf area and weight could in part be due to developmental delays because all three lines, but especially *AtPROPEP6:2-oe,* were delayed in the onset of bolting compared to wild-type controls. The combined results of our bioassays and morphological measurements indicate that *AtPROPEP6* impacts both nematode infection and plant growth, but the effects of *AtPROPEP6* overexpression on nematodes do not seem to be correlated with or dependent upon gross morphological changes in the plant. Rather, the effects of *AtPROPEP6* overexpression are likely attributed to enhanced immune signaling.

Although Pep–PEPR signaling relies on a broadly conserved receptor-based recognition mechanism, apart from a few C-terminal residues that mediate binding to the LRR domain of PEPR receptors.^42^ The remaining non-conserved regions are highly disordered, making it difficult to define clear orthologous relationships across species. Peps exhibit striking sequence divergence. Even within *Arabidopsis*, individual paralogs share only 12–47% sequence identity.^6^ Consequently, Pep paralogs often carry out distinct and non-redundant biological functions.^43^ Furthermore, Pep-PEPR signaling represents highly species-specific communication, which is supported by experiments showing that *A. thaliana* PEPR1 cannot perceive maize *Zm*PEPs, and vice versa.^43^ Hence, a comprehensive understanding of the Pep–PEPR system requires independent functional characterization of individual paralogs and putative orthologs, as their distinct biological roles cannot be inferred from sequence alone. Thus, characterizing *AtPROPEP6* in the context of nematode stress and impact on plant morphology provides a meaningful step toward deciphering the complexity of this signaling network.

## Supplementary Material

SUPPLEMENTARY MATERIALS.docx SUPPLEMENTARY MATERIALS.docx

## Data Availability

Data that support the findings of this study are available from the corresponding author upon reasonable request.
