# Supplementary material for: Overexpression of AtPROPEP6 enhances Arabidopsis thaliana resistance to Southern root-knot nematode Meloidogyne incognita
Source: Plant Signal Behav. 2026 Feb 8;21(1):2624239. doi: 10.1080/15592324.2026.2624239 (PMC12893677; doi:10.1080/15592324.2026.2624239)
Supplement: SUPPLEMENTARY MATERIALS.docx [file KPSB_A_2624239_SM6881.docx]

**SUPPLEMENTARY MATERIALS**

**Table S1. Primers used for qRT-PCR**

| Name | Gene accession # | Sequence (5’ to 3’) | Reference | Annealing temp | Primer pair efficiency |
| --- | --- | --- | --- | --- | --- |
| Actin2_F | At3G18780 | GTATCGCTGACCGTATGAG | Tomaz et al., 2010 | 52°C | 98% |
| Actin2_R |  | CTGCTGGAATGTGCTGAG |  |  |  |
| EF-1a_F | At5G60390 | TGAGCACGCTCTTCTTGCTTTCA | Czechowski et al., 2005 | 57°C | 89% |
| EF-1a_R |  | GGTGGTGGCATCCATCTTGTTACA |  |  |  |
| F-BOX_F | At5G15710 | GGCTGAGAGGTTCGAGTGTT | Lilly et al., 2011 | 54°C | 86% |
| F-BOX_R |  | GGCTGTTGCATGACTGAAGA |  |  |  |
| PDF1.2_F | At5G44420 | ATGGCTAAGTTTGCTTCCA | This study | 50°C | 93% |
| PDF1.2_R |  | TTAACATGGGACGTAACAGATAC |  |  |  |
| PR1_F | At2G14610 | CTCATACACTCTGGTGGG | This study | 50°C | 96% |
| PR1_R |  | TTGGCACATCCGAGTC |  |  |  |
| PEP6_F | At2G22000 | ATGGAAGTTAATGGAGAAGA | This study | 50°C | 97% |
| PEP6_R |  | ATTGTTTTGACCAGGTCGT |  |  |  |
| ACPLA1_F | At1G69900 | GGAATATTTCCATCGCCGATAC | Safaeizadeh et al., 2024 | 58°C | XX% |
| ACPLA1_R |  | GATCCTGGGTCACTTGTATCAG |  |  |  |
| PR4_F | At3G04717 | TGTTCTCCGACCAACAACTG | Hamamouch et al., 2010 | 57°C | XX% |
| PR4_R |  | CAATGAGATGGCCTTGTTGA |  |  |  |
| PP22B13_F | At1G56240 | CGTGACACAGACTAAATAATAGATC | Safaeizadeh et al., 2024 | 55°C | XX% |
| PP22B13_R |  | CCTCTGAAATAGGGATCAAGATG |  |  |  |

**Table S2. Defense-related Cis-Regulatory Elements (CREs) in the promoter of the *AtPROPEP6* gene (1,743 bp upstream from the transcription start site +1).**

| **name and NewPLACE code** | **sequence^a^** | **n^b^** | **position (strand)** | **Species** | **function** |
| --- | --- | --- | --- | --- | --- |
| BIHD1OS S000498 | TGTCA | 2 | 314 (+), 439 (+) | *Oryza sativa* (rice) | Disease resistance responses |
| SEBFCONSSTPR10A S000391 | YTGTCWC | 1 | 884 (-) | *Solanum tuberosum* (potato) | Silencing element in PR10a gene promoter |
| MYB1LEPR S000443 | GTTAGTT | 1 | 680 (-) | *Arabidopsis thaliana*; *Lycopersicon esculentum* (tomato) | Defense-related gene expression |
| GT1GMSCAM4 S000453 | GAAAAA | 3 | 1,096 (-), 1,362 (-), 1,426 (-) | *Glycine max* (soybean) | GT-1 transcription factor binding site, pathogen and salt induction |
| RAV1BAT S000315 | CACCTG | 2 | 630 (+), 1,655 (-) | *Arabidopsis thaliana* | Plant disease resistance |
| WRKY71OS S000447 | TGAC | 8 | 50 (-),  55 (-),  315 (-),  440 (-),  712 (+),  992 (-),  1,107 (+) | *Oryza sativa* (rice); *Petroselinum crispum* (parsley) | Binding site of rice WRKY71 which is a transcriptional repressor of the GA signaling pathway |
| WBOXNTERF3 S000457 | TGACY | 5 | 49 (-),  54 (-),  712 (+),  1,107 (+),  1,662 (-) | *Nicotiana tabacum* (tobacco) | Activation of ERF3 gene transcription by wounding |
| WBBOXPCWRKY1 S000310 | TTTGACY | 1 | 1,662 (-) | *Arabidopsis thaliana*; *Petroselium crispum* (parsley) | Elicitor response elements in the promoters of parsley PR1 gene |
| WBOXATNPR1  S000390 | TTGAC | 3 | 55 (-),  315 (-),  1,663 (-) | *Arabidopsis thaliana* | Salicylic acid (SA)-induced WRKY DNA binding protein |
| WBOXNTCHN48  S000508 | CTGACY | 1 | 49 (-) | *Nicotiana tabacum* (tobacco) | Elicitor-responsive  transcription of defense genes in tobacco |
| ASF1MOTIFCAMV  S000024 | TGACG | 1 | 991 (-) | *Nicotiana tabacum* (tobacco); *Arabidopsis thaliana* | Biotic and abiotic stress |
| ELRECOREPCRP1  S000142 | TTGACC | 2 | 54 (-),  1,662 (-) | *Petroselinum crispum* (parsley)*; Nicotiana tabacum* (tobacco) | Elicitor response elements in the promoters of parsley PR1. pathogen- and wound-induced signaling |

**
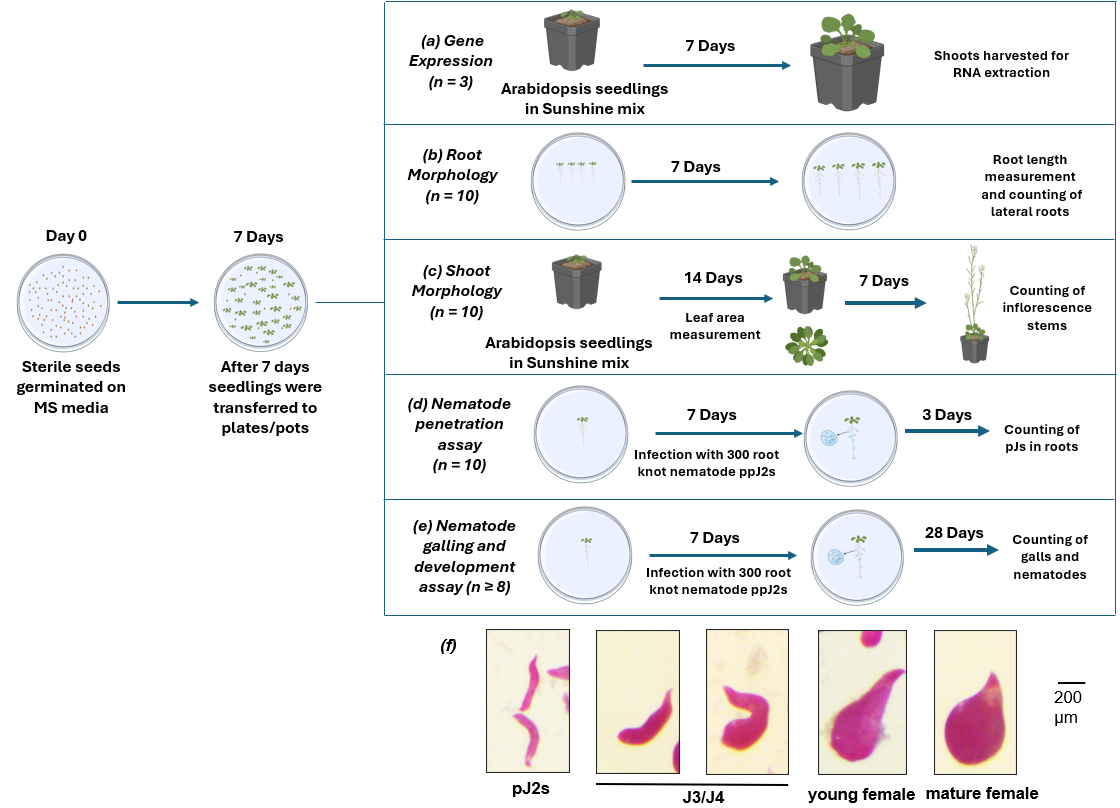
**

**Figure S1. Schematic representation of the experimental design used to evaluate the effect of *AtPROPEP6* overexpression**. **(a)** Gene expression analysis in transgenic *AtPROPEP6* overexpression lines. **(b)** Root morphology analysis. **(c)** Shoot phenotypic analysis of *AtPROPEP6* plants. **(d)** Root-knot nematode penetration assay **(e)** Root-knot nematode galling and development assay. **(f)** Developmental stages observed in bioassays. Pre-parasitic J2s (ppJ2s), parasitic J2s (pJ2s) – vermiform swollen, third stage (J3) to fourth stage (J4) - sausage shaped, young female - ovoid, mature female - globose (Velloso et al., 2022). n represents the number of biological replicates per experiment.

**
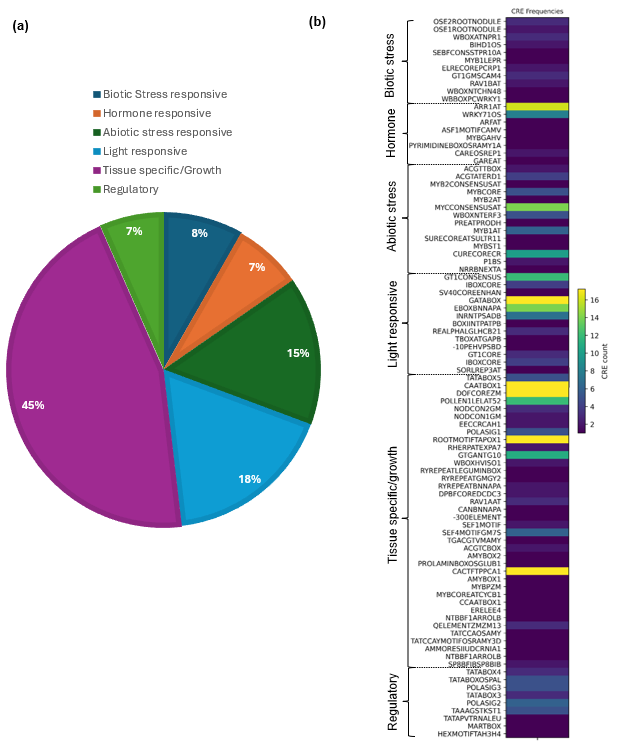
**

**Figure S2. Cis-Regulatory Elements (CREs) in the promoter of the *AtPROPEP6* gene.** **(a)** Pie chart representing the distribution of CREs categories. **(b)** Heatmap showing the frequency and categories of CREs. Color intensity reflects the number of occurrences of each CRE, with darker colors indicating lower frequencies and bright yellow indicating the highest. *AtPROPEP6* promoter was defined as a 1,743 kb region upstream from the translation start site 1+ (which represents the entire intragenic region up to the next 5’ open reading frame) and analyzed by using NewPLACE (Higo et al., 1999).


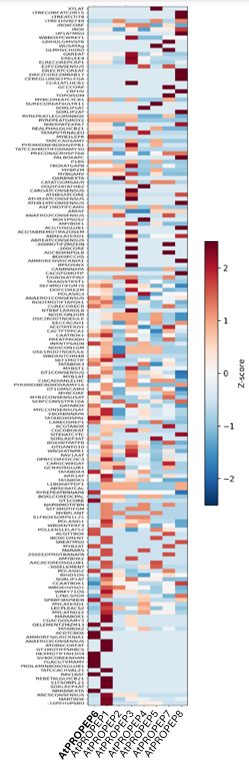


**Figure S3. Heatmap showing cis-regulatory elements (CREs) identified in the promoter regions of *AtPROPEP* genes.** Promoters were defined as the upstream region extending from the translation start site (+1) to the beginning of the adjacent open reading frame of the neighboring gene and were analyzed using NewPLACE (Higo et al., 1999). Values represent z-scores calculated from raw CRE counts across *AtPROPEP1–AtPROPEP8* (Cheadle et al., 2003). Red colors indicate relative enrichment, whereas blue colors indicate relative depletion. Z-scores were clipped to ±3 for visualization.

**
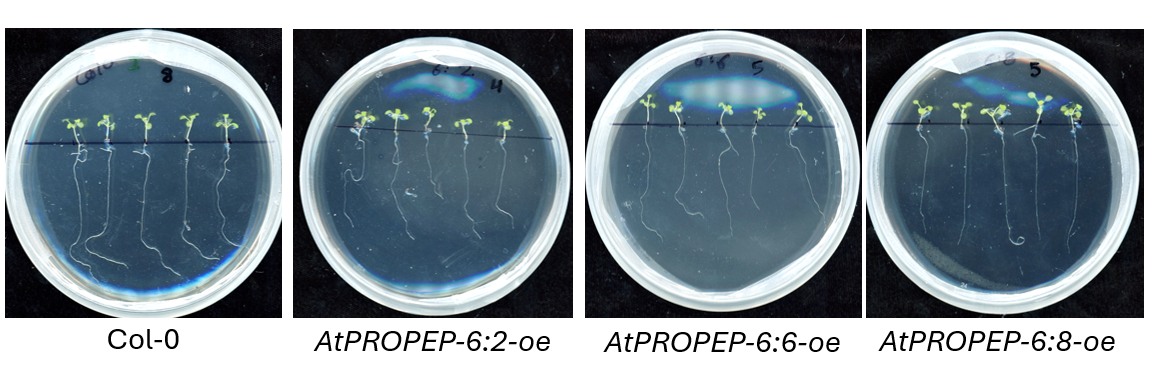
**

**Figure S4. Representative photographs of roots from the *AtPROPEP6* overexpression lines.** Photographs of roots in a control wild-type Col-0 and homozygous transgenic *AtPROPEP6-oe* lines (6:2, 6:6, and 6:8) were taken at 14 days of growth, immediately before length measurements and lateral root scoring.


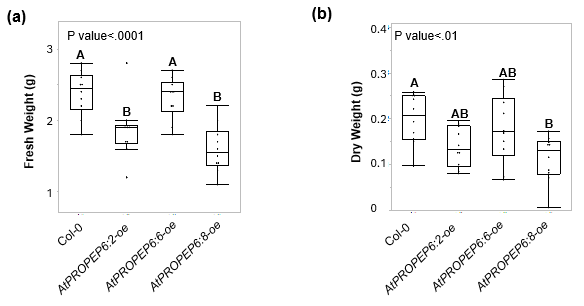


**Figure S5. Constitutive overexpression of *AtPROPEP6* impacts leaf weight.** **(a)** Fresh weight and **(b)** dry weight of leaves in a control wild-type Col-0 and homozygous transgenic *AtPROPEP6-oe* lines (6:2, 6:6, and 6:8). Different letters indicate significant differences (one-way ANOVA with Tukey's HSD, n = 10).
